# Characteristics of the memory sources of dreams: A new version of the content-matching paradigm to take mundane and remote memories into account

**DOI:** 10.1371/journal.pone.0185262

**Published:** 2017-10-11

**Authors:** Raphael Vallat, Benoit Chatard, Mark Blagrove, Perrine Ruby

**Affiliations:** 1 Lyon Neuroscience Research Center (CRNL), Brain Dynamics and Cognition Team (DYCOG), INSERM UMRS 1028, CNRS UMR 5292, Université Claude Bernard Lyon 1, Université de Lyon, Lyon, France; 2 Swansea University, Sleep laboratory, Swansea, Wales, United Kingdom; Associazione OASI Maria SS, ITALY

## Abstract

Several studies have demonstrated that dream content is related to the waking life of the dreamer. However, the characteristics of the memory sources incorporated into dreams are still unclear. We designed a new protocol to investigate remote memories and memories of trivial experiences, both relatively unexplored in dream content until now. Upon awakening, for 7 days, participants identified the waking life elements (WLEs) related to their dream content and characterized them and their dream content on several scales to assess notably emotional valence. Thanks to this procedure, they could report WLEs from the whole lifespan, and mundane ones before they had been forgotten. Participants (N = 40, 14 males, age = 25.2 ± 7.6) reported 6.2 ± 2.0 dreams on average. For each participant, 83.4% ± 17.8 of the dream reports were related to one or more WLEs. Among all the WLEs incorporated into dreams dated by the participants (79.3 ± 19%), 40.2 ± 30% happened the day before the dream, 26.1 ± 26% the month before (the day before excluded), 15.8 ± 21% the year before the dream (the month before excluded), and 17.9 ± 24% happened more than one year before the dream. As could be expected from previous studies, the majority of the WLEs incorporated into dreams were scored as important by the dreamers. However, this was not true for incorporated WLEs dating from the day before the dream. In agreement with Freud’s observations, the majority of the day residues were scored as mundane. Finally, for both positive and negative WLEs incorporated into dreams, the dreamt version of the WLE was rated as emotionally less intense than the original WLE. This result, showing that dreams tend to attenuate the emotional tone of waking-life memories towards a more neutral one, argues in favor of the emotional regulation hypothesis of dreaming.

## Introduction

A way to progress in our understanding of the possible functions of dreaming is to investigate the parameters constraining or influencing dream content. An extensive amount of research has investigated the memory sources of dreams and has demonstrated that dream content very rarely replays an episodic memory as it is remembered [1,2], although it is often related to some elements of the waking life of the dreamer (for reviews [3–5]). This has led to the so-called “continuity hypothesis of dreaming” which simply states that dreams reflect waking life experiences [6]. In 2010, Michael Schredl reported that according to results from studies using various methods (assessing temporal references of dream elements retrospectively, experimental manipulation of the pre-sleep situation, field studies, fluctuations over time within persons, differences between persons), the five following factors influence the incorporation of a waking life element (WLE) into dreams:

- Time (the more remote the WLE, the less incorporated into dreams, e.g. [7,8])
- Emotion intensity (the more emotional the WLE, the more incorporated into dreams, e.g. [9,10])
- Type of experience (e.g. working with a computer is less often incorporated into dreams than is chatting with friends, e.g. [11])
- Personality traits of the dreamer (field dependence and thin boundaries may moderate the magnitude of the continuity between waking and dreaming, e.g. [12])
- Time of the night (dreams of the second part of the night comprise more elements of the distant past, while dreams of the first part of the night incorporate mostly recent daytime experiences, e.g. [13,14]).

However some characteristics of dream content do not fit with this modelling of the data. Firstly, some body injuries—be it congenital or acquired—such as amputation, paraplegia and deafness, are less incorporated into dream reports than this model would predict considering how highly emotional and central to the person’s life they are [15–17]. Secondly, the available results show that WLEs incorporated into dreams are more emotionally intense than are WLEs that are not incorporated into dreams [9,10], while day residues (WLEs from the day before the dream), which are known to be a great part of the WLEs incorporated into dreams [18–23], are often mundane, as noticed by Freud [24].

Even if they are not necessarily contradictory with Freud’s observation, results showing a bias towards the incorporation of emotional elements into dreams may be explained by the method used in experimental studies so far, i.e. the content matching paradigm. It requires the participants to rate a posteriori (i.e. at the end of the 14 days of the experiment) similarities between a day diary and a dream diary completed for 14 days [9,10]. Such a method has the advantage of controlling for retrospective availability of memories for elements when participants relate dream content to WLEs, but it has the drawback of missing mundane experiences that are not recorded in the diary. As a consequence, previous studies could not assess whether mundane WLEs were incorporated into dreams.

The present study aims to investigate more exhaustively than previously achieved the characteristics of the WLEs incorporated into dreams, notably by assessing their remoteness on a life-time scale and by taking mundane WLEs into account. To do so, instead of asking dreamers to keep a day diary, we asked participants to report and characterize the WLEs related to their dreams immediately upon awakening. This strategy presents several advantages regarding previous methods. Firstly, any remembered WLE at any timescale can be considered. This method offers then the possibility of investigating the incorporation of WLEs across the whole life span, which has been rarely attempted until now [8,25]. Secondly, as the reported memory sources of a dream are dependent on the delay between the dream and the task to report memory sources, the sooner the task after the dream, the more chances we have to identify the true memory sources of the dream [26]. Thirdly, as the connections between elements of waking life and dream content are assessed when the memories of the preceding days are still fresh, this method enables the recall of trivial WLEs from at least the few days before the dream. Using this new approach, we are able to test whether emotional WLEs are still preferentially incorporated into dream reports when trivial WLEs are taken into account and to investigate the emotionality and significance of WLEs incorporated into dreams as a function of their remoteness. The results will be discussed regarding previous literature and current hypotheses about dream function, notably those attributing a role to dreaming in emotion regulation [27,28] and memory consolidation [29–31].

## Material and methods

### Participants

An announcement describing the study was sent by emails to students and staff of Lyon University. The inclusion criteria were (1) a habitual dream recall frequency of at least 3 mornings per week with a dream in mind, (2) the agreement for reporting all recalled dreams exhaustively without censorship and (3) the agreement for sleeping at habitual hours without excessive consumption of alcohol during the 7 days of the experiment. Forty participants (14 males, age = 25.2, SD = 7.6) were recruited accordingly. The study conformed with French regulation and ethics regarding research in humans and approval was granted by the “Centre National de Recherche Scientifique, Cellule Informatique et Liberté”. Subjects gave written informed consent according to the Declaration of Helsinki and were paid for their participation.

### Procedure and questionnaires

The recruited participants were asked to come to the lab for a 30 minutes meeting. At this occasion, the experimenters described the study requirements, gave instructions and answered any questions of the participants. Experimenters explained that the aim of the study was to identify the possible links between waking life experiences and dream content and that participants were expected to report without censorship any kind of waking life elements related to their dreams (episodes, objects or characters, whether recent or old, important or trivial).

The participants were also given a questionnaire designed for the study that they had to fill in before starting reporting their dreams. This questionnaire asked about various aspects of the participant’s lives (age; gender; habitual dream recall frequency; habitual sleep time and duration; education; work; first names of siblings, parents, children, current and previous partners, closest friends and any deceased friends or family members; hobbies; possible experiences with a high emotional load in the last 4 weeks; a list of the most personally important places of habitation or vacation; a list of the most important current concerns). This questionnaire was designed to identify the personally important aspects of the participant’s life, to enable us to score their possible appearances in dream content.

Next, participants were requested to report their dreams using a voice recorder immediately upon awakening for seven consecutive days. They were asked to describe their dream content in as much detail as possible without adding interpretations. After each dream was reported participants completed a questionnaire about possible links between the dream content and their waking life. Participants had to tell whether they felt that parts of their dream were obviously related to some features of their waking lives. It was made clear during the initial interview that any kind of waking life feature could be considered (e.g., places, characters, actions, events, objects, thoughts) even if trivial. If so, for each link that was made the participant had to: 1) score the emotional valence of the element of the dream that reminded them of an element of their waking life, 2) describe the waking life element incorporated into their dream with written words, dating it when possible and rating it on various scales (from 1 to 10) to assess its familiarity, frequency, emotional valence, importance, personal versus professional dimension, social dimension, how much a concern it was, and how similar it was to the corresponding dream content. If several waking life elements were incorporated into a dream, participants had to describe and rate each of them separately.

The questionnaire also asked participants to quantify several aspects of dream content. Dreamers had to: 1) rate the emotional intensity (scales from 1 to 10) and the emotional valence (scale from 1 very negative, to 10 very positive) of the dream and to report the emotions encountered during the dream (primary emotions, i.e. joy, sadness, fear, anger, disgust, surprise were proposed as possible answers as well as a blank field for other possible emotions), 2) count the number of characters in the dream, and report for each character whether they are familiar in their waking life (and if so, their name or relationship to the participant), unknown in their waking life, or with mixed characteristics, 3) report the number of places in the dream and whether these places are familiar in their waking life (and if so, their name), unknown in their waking life or with mixed characteristics. This questionnaire was designed for the study with the objective of complying with two contradictory aims: minimizing the time to fill in the questionnaire each morning and maximizing the quality and quantity of information on the dream and on the WLEs related to the dream. A preliminary study with 10 subjects was used to optimize the questionnaire (those subjects were not included in the main study). At the end of the experiment participants were asked whether they had experienced a strongly emotional event or new concern during the 7 days of the experiment.

Note that the main aim of the study was to identify the temporal and emotional characteristics (among others) of the WLEs incorporated into dreams. We did not aim to investigate the memory type of the WLEs reported (episodic, autobiographic and semantic). Future studies will be needed to investigate the interaction between the factors we studied and memory type.

### Data analysis and scoring of dream content

#### Word count

Audio reports were transcribed from audio to written language by the company TranscribeMe! (http://transcribeme.com/) and were subsequently checked by the experimenters before word count.

#### Characters and settings

Crowds counted as one person if individuals were not explicitly specified, following the Hall & Van de Castle rating system [32]. Among the existing characters, close family members and close friends were counted by RV and BC using the names mentioned *a priori* in the initial questionnaire. Similarly, among the known places, RV and BC counted the significant ones, defined as those mentioned in the initial questionnaire by the participants. RV and BC conducted scorings independently and, in case of disagreement between scores, reached a consensus after discussion.

#### Emotional valence and intensity

For each dream the emotional valence of the WLE(s) incorporated into the dream and the emotional valence of the dream were compared. If several WLEs were incorporated into one dream their average valence was considered.

The emotional intensity of WLEs incorporated into dreams was derived from the rating of emotional tone by a transposition of the initial valence scale (1 = very negative, 10 = very positive) to an intensity scale of zero to four (0, neutral; 1, feebly intense; 4, very intense; irrespective of the positive or negative tone of the WLE). We considered as low emotional intensity the ratings 4, 5 or 6 on the 1-to-10 scale. Medium intensity was attributed to ratings of 2, 3, 7 or 8 and high intensity to a rating of 1 or 9–10. In other words, positive and negative WLE were re-rated on a 1 to 4 scale where 1 means feeble positive/negative and 4 means very positive/negative.

#### Temporal remoteness of WLEs incorporated into dreams

First, to assess the predominant temporal origin of the WLEs incorporated into dreams, we compared the proportion of WLEs in each of the 3 following categories: day before (day-residues), month before (day before the dream excluded) and older than one month. Secondly, to test whether we reproduce the dream lag effect (i.e. as compared to what would be expected according to memory decay with time, some studies reported an overrepresentation in dreams of the WLEs that happened approximately one week before the dream), we compared the proportion of WLEs in each of the 4 following categories: day before, 2 to 5 days before, 6 to 9 days before and 10 to 14 days before the dream. We chose for the analysis temporal categories according to the time scale of the maximum of the effect previously reported (6 days before the dream, [22]; 7 days before the dream, [21,23]; 9 days before the dream, Jouvet [33] quoted in [34]), and of equal durations (4 days), i.e. WLEs that happened 2 to 5 days and 6 to 9 days before the dream.

#### Concerns

Concerns listed in the initial questionnaire were grouped into 7 thematic categories (Work/Study, Family/Friends, Leisure (i.e., extra-professional or extra-scholar activity), Everyday life, Romantic relationship, Self-related and Health) by RV and BC. The assessment of whether or not a concern listed in the initial questionnaire was incorporated into a dream was not done at awakening by the participants but *a posteriori* by two scorers. Scorers used the concerns listed in the initial questionnaire to assess whether the WLEs incorporated into dreams could be considered as part of the current concerns of the dreamers. RV and BC conducted scorings independently and, in case of disagreement between scores, reached a consensus after discussion.

#### Re-occurrence of a WLE in several dreams

As it has been shown that some memories may be iteratively processed across subsequent nights (e.g. [35]), for all participants, RV and BC assessed whether some WLEs were incorporated into several dreams during the time of the experiment (in case of disagreement, they reached a consensus after discussion).

#### Statistics

Except when specified, values reported are always grand means (mean of each participant’s mean) and standard deviation. Statistical testing were made using repeated measures ANOVA, followed by Tukey HSD pairwise comparisons in case of significance (alpha level = 0.05).

## Results

A total of 247 dreams were reported, ranging from 16 to 3691 words (average per participant, 543 ± 262 words). On average the participants reported 6.2 ± 2 dreams in the 7 days of the experiment (range = 3–14; 8 participants reported at least once, more than one dream per morning, leading to 14 mornings with multiple dream reports). Among all the dreams reported 207 have been related to a WLE (83.8%). For each participant, 1) on average, 83.4% ± 17.8 of their dream reports were related to one or more WLEs, 2) on average 1.8 ± 1.6 incorporations per dreams were reported (2.1 ± 1.5 considering only dream reports with incorporation of WLE, maximum per dream = 19). Dream reports for which dreamers reported a link with one or several WLEs were in average longer than dream reports that were not related to WLEs (576.0 ± 280.1 vs 411.9 ± 118.4 words; paired t-test = 0.04). S1 Table provides examples of WLEs incorporated into dreams.

### Emotions in the dreams

The average emotional intensity of the reported dreams (scale from 1, low to 10, high) was 5.65 ± 1.55. In average, for each subject 30.1% ± 27.2 of the dreams were of low emotional intensity (score inferior to 5), 13.5 ± 16.6 were neutral (score equal to 5) and 56.4 ± 29.6 were of high emotional intensity (score superior to 5). The average emotional valence of the reported dreams (scale from 1, highly negative to 10, highly positive) was 4.79 ± 0.91. In average, for each subject 42.7 ± 27.2 of the dreams were negative (score inferior to 5), 30.4 ± 24.7 were neutral (score equal to 5) and 26.9 ± 20.2 were positive (score superior to 5). The mean number of emotions per dream was 1.82 ± 0.69. The most frequent emotion reported by participants was surprise (present in 29.96% of the dream reports), followed by fear (26.72%), joy (25.10%), anger (20.65%) and sadness (16.59%).

**Fig 1:**
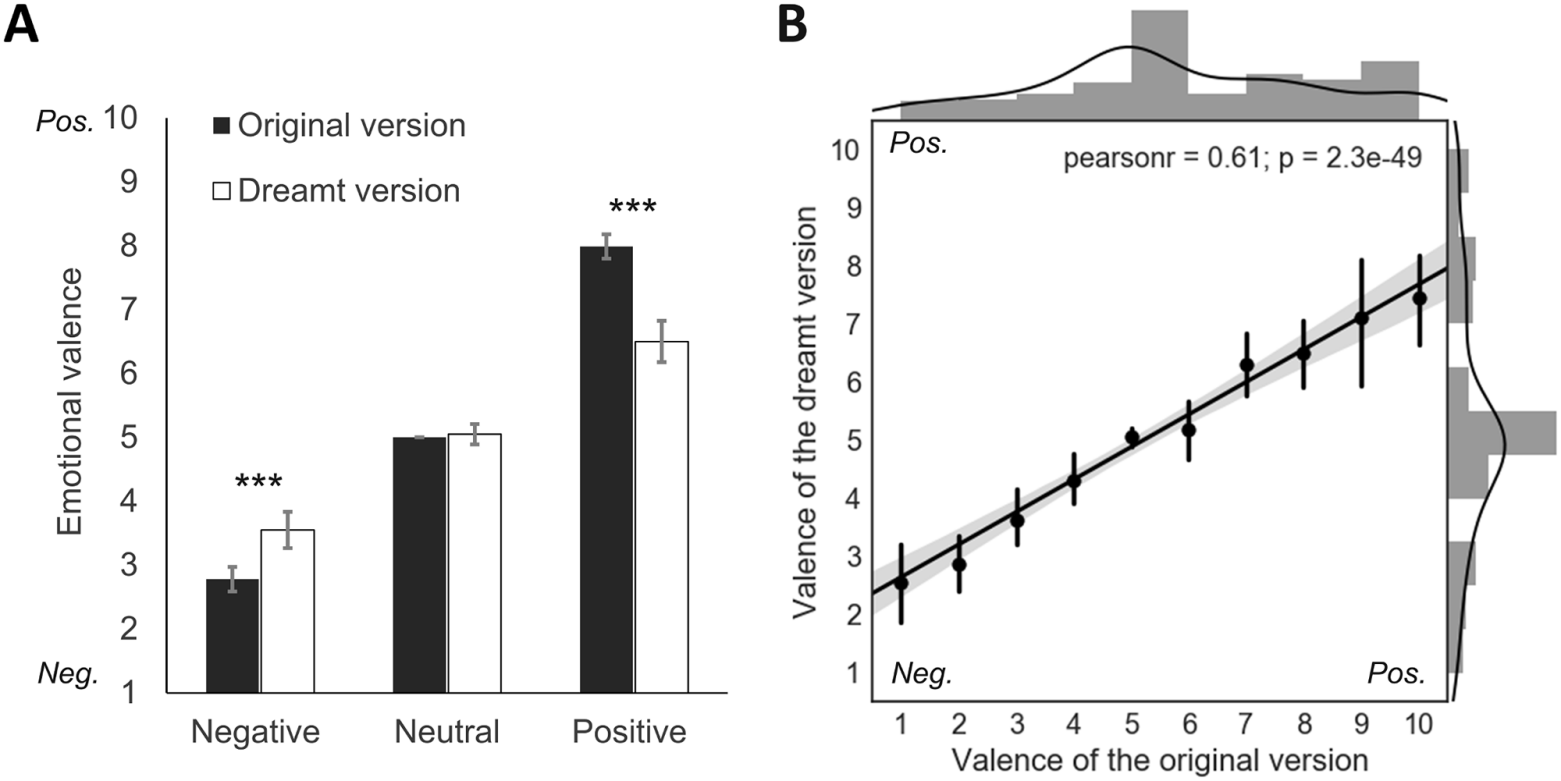
Emotional valence of the dreamt version and the original version of the WLEs incorporated into dreams. A) Emotional valence of the dreamt version (white bars) and the original version (black bars) of the WLEs incorporated into dreams for positive (rating > 5), neutral (rating = 5) and negative (rating < 5) elements. The dreamt version was scored as more neutral than the original version of the WLEs. B) Significant positive correlation between the emotional valence ratings of the original and the dreamt versions of the WLEs incorporated into dreams. Gray bars indicate the distribution of the ratings for the original and the dreamt version of the WLEs incorporated into dreams. Error bars represent 95% confidence intervals. *** p <.001.

A t-test revealed that WLEs incorporated into dreams were rated as significantly more positive (5.61 ± 2.2) than the dreams in which they had been incorporated into (4.81 ± 1.9; paired t-test p <.001, n = 247). Moreover, in average WLEs incorporated into dreams were rated as significantly more positive (5.74 ± 2.4,) than the element of the dream related to that WLE (5.30 ± 2.2; paired t-test on the emotional ratings of the WLE and the ones of the dreamt version of the WLE, p < .001, n = 492). Interestingly, when we ran the analysis separately for positive, neutral and negative WLEs (Fig 1A), we found that positive WLEs (valence > 5) were rated as more positive (8.0 ± 1.4) than the element of the dream related to that WLE (6.5 ± 2.4; paired t-test p < .001, n = 214), and that negative WLEs (valence < 5) were rated as more negative (2.78 ± 1.1) than the element of the dream related to that WLE (3.5 ± 1.6; paired t-test p <.001, n = 129). For neutral WLEs we found no significant difference between the valence of the WLE (valence = 5) and the valence of the element of the dream related to that WLE (5.05 ± 1.0; paired t-test p = .53, n = 140). A correlation analysis further showed the grouping of the emotional valence grades toward the 5 (neutral) grade for the dreamt version of the WLE as compared to its original one (Pearson, r = 0.61, p < .001) (Fig 1B).

### Places and characters of the dreams

**Fig 2:**
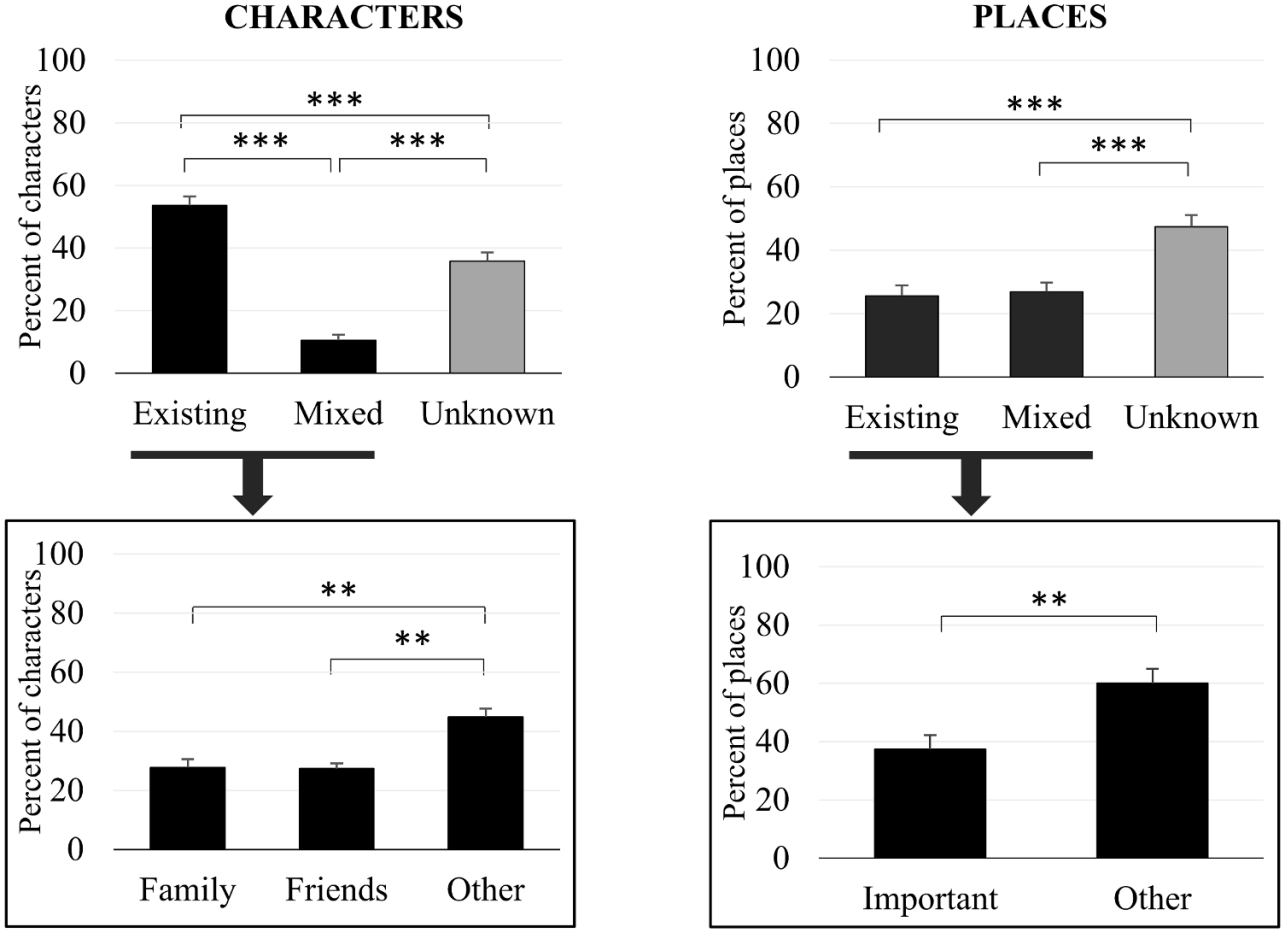
Characters and places of the dreams. Error bars represent standard error of the mean. **p<.*01* ***p<.*001*.

The mean number of characters per dreams was 4.7 ± 2.3. At least one character was reported in 96.55% of the dream reports. As illustrated in Fig 2, the majority of the dreamed characters existed in waking life (54% ± 18), and 11% ± 11 had mixed attributes (e.g. in the dream the dreamer knows that a character is someone that he knows even if he/she has modified physical attributes, or the other way around). About one third of the dreamed characters were unknown to the dreamer (36% ± 18). Among the existing characters, close family members and close friends (i.e. those mentioned *a priori* in the initial questionnaire) accounted each for 28% ± 20 and 27% ± 20 of the dreamt characters. The remaining (45% ± 25) included existing but less close persons such as distant relatives, distant friends and work colleagues.

There was an average of 2.3 ± 1.5 places per reported dream. Nearly half (47% ± 23) of the dreamed places were unknown to the participant and the remaining places were either existing (26% ± 10) or with mixed attributes (27% ± 18). Among the existing places, 38% ± 30 had been reported as significant in the initial questionnaire by the participants (Fig 2).

### Incorporation of the current concerns of the dreamer into the dreams

On average participants listed 4.7 ± 1.2 concerns in the initial questionnaire. A great majority of the participants (70%) incorporated at least one waking life element related with their current concerns (i.e. those listed in the initial questionnaire) during the 7 days of the experiment. In average, 23% ± 21 of the dream reports of each subjects incorporated a current concern. Finally, for each subject among all the concerns listed in the initial questionnaire an average of 25% ± 22 were incorporated into a dream report during the 7 days of the experiment (see Table 1 for examples). The category with the greatest number of concerns happened to be the Work/Study one. The distribution of concerns according to each category and the percentages of concerns in each category incorporated into dreams are illustrated in S1 Fig.

**Table 1:** Examples of concerned incorporated into a dream.

Subject	Concern	Dream report
S29	Concern n°1: *“My aunt passed away recently and I miss her terribly*. *I know she will not come back*, *but some days I still hope it will happen*.*”*	Day 6: *“I descend into another world to pick up my aunt and realize that it is not possible—I feel a deep anguish and I am cold”*
S4	Concern n°1: *“My girlfriend suffers from anorexia*. *She is much stressed and vomits almost everything she eats*. *I am trying to help her relax and regain self-confidence*.*”*	Day 5: *“My girlfriend threw up what she had eaten after a big meal*. *I comfort her*.*”*
S28	Concern n°2: “*To succeed in my university studies”*	Day 1: *“It was the day of publication of the 1st semester’s results*. *We were in the tramway*. *The teachers were within the tramway (which was also the classroom)*. *I was waiting for the teachers to tell us our results*. *But nobody told us and they just gave us the corrections instead*. *I felt very impatient*.*”*

### Re-occurrence of a WLE in several dreams

Re-occurrence of a WLE in several dreams was found for only 6/40 participants and only one of them mentioned explicitly that it was a re-occurrence. For all six we found a WLE incorporated in two different dreams and, for one of them, a WLE incorporated in 3 different dreams. The WLEs re-occurring in several dreams were related to current or emotional concerns (e.g. preparation of a journey abroad, awaiting the results of university exams, grand-mother in hospital for a recent accident, fear of succumbing to a morally reprehensible desire, meeting with the new boyfriend of his ex-girlfriend) or to recent hobbies (a TV show, a role play, hiking with friends).

### Characteristics of WLEs incorporated into the dreams

#### Average scores

**Fig 3:**
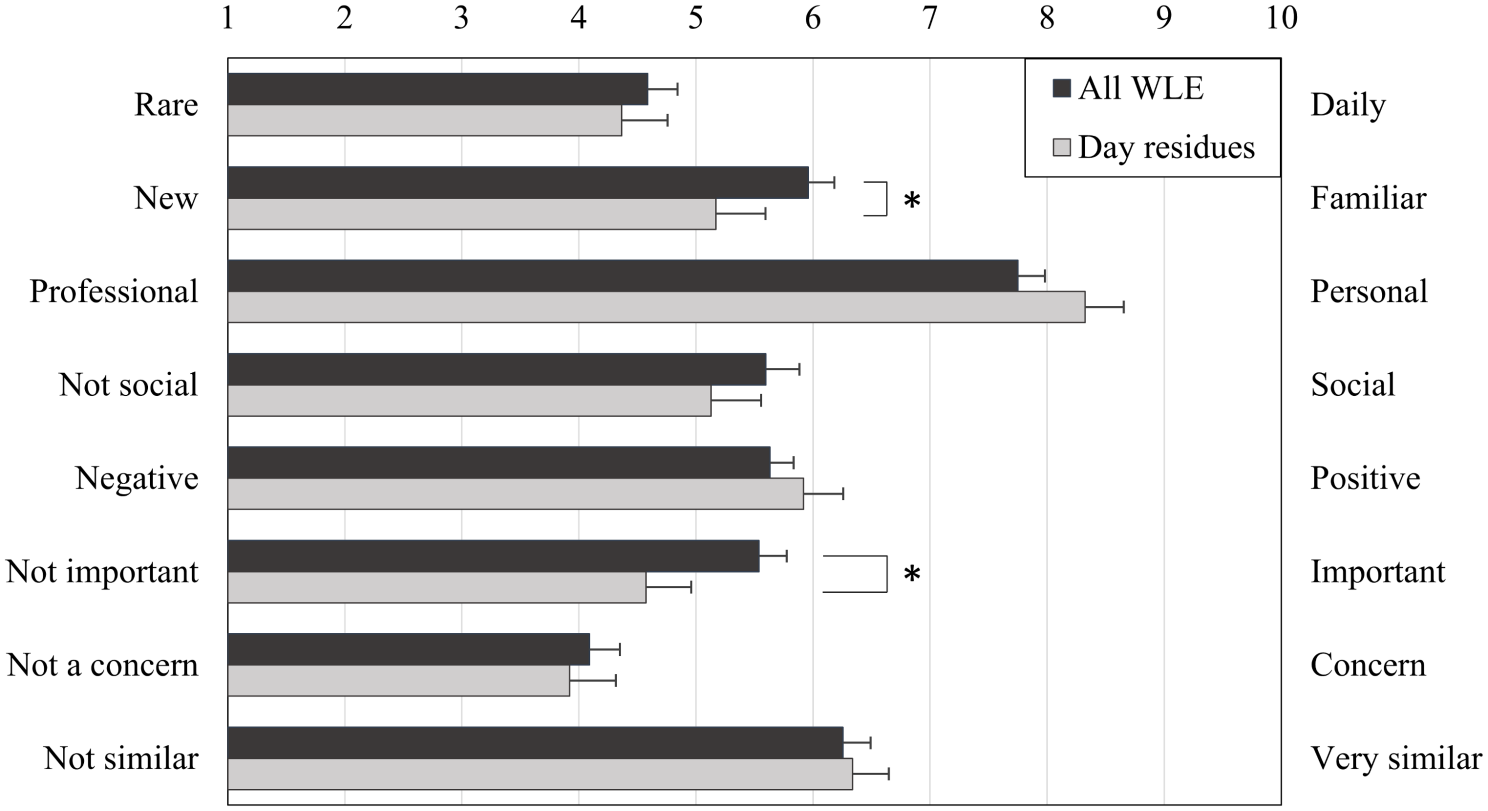
Characteristics of waking life elements incorporated into dreams. All waking life elements (black) and day residues only (grey). Error bars represent standard error of the mean. *p<.*05*.

The averages scores given by the dreamer at awakening for various dimensions of the WLEs incorporated into their dream reports are presented in Table 2 and Fig 3. T-tests revealed that as compared to all WLEs, day-residues were scored as less familiar, less important and tended to be scored as less emotionally intense. The distribution of WLEs with a rating inferior, equal and superior to 5 can be found in S1 File and S2 Fig.

**Table 2:** Averaged scores given by the dreamers at awakening to describe the WLEs incorporated into their dreams.

Characteristics	All WLEs	Day-residues	*p-value*
Frequency *(1*: *Rare—10*: *Daily)*	4.5 ± 1.5	4.4 ± 2.2	*0*.*6*
Familiarity *(1*: *New—10*: *Familiar)*	6 ± 1.5	5.2 ± 2.4	*0.*03*
Personal dimension *(1*: *Professional—10*: *Personal)*	7.8 ± 1.4	8.3 ± 1.9	*0*.*2*
Social dimension *(1*: *Not social—10*: *Social)*	5.7 ± 1.8	5.1 ± 2.4	*0*.*2*
Emotional valence *(1*: *Negative—10*: *Positive)*	5.7 ± 1.3	5.9 ± 1.9	*0*.*1*
Emotional intensity *(0*: *None—4*: *Very intense)*	1.9 ± 0.8	1.6 ± 1.2	*0*.*08*
Importance *(1*: *Not important—10*: *Important)*	5.5 ± 1.5	4.6 ± 2.2	*0.*01*
Current concern *(1*: *Not a concern—10*: *Concern)*	4.1 ± 1.6	3.9 ± 2.2	*0*.*9*
Similarity with dream *(1*: *Not similar—10*: *Identical)*	6.3 ± 1.5	6.3 ± 1.7	*0*.*6*

Scores for all WLEs and day-residues only are presented (*p-values* of the t-test comparing the scores in the two categories are presented).

#### Proportions of the WLEs incorporated into dreams as a function of their temporal remoteness

**Fig 4:**
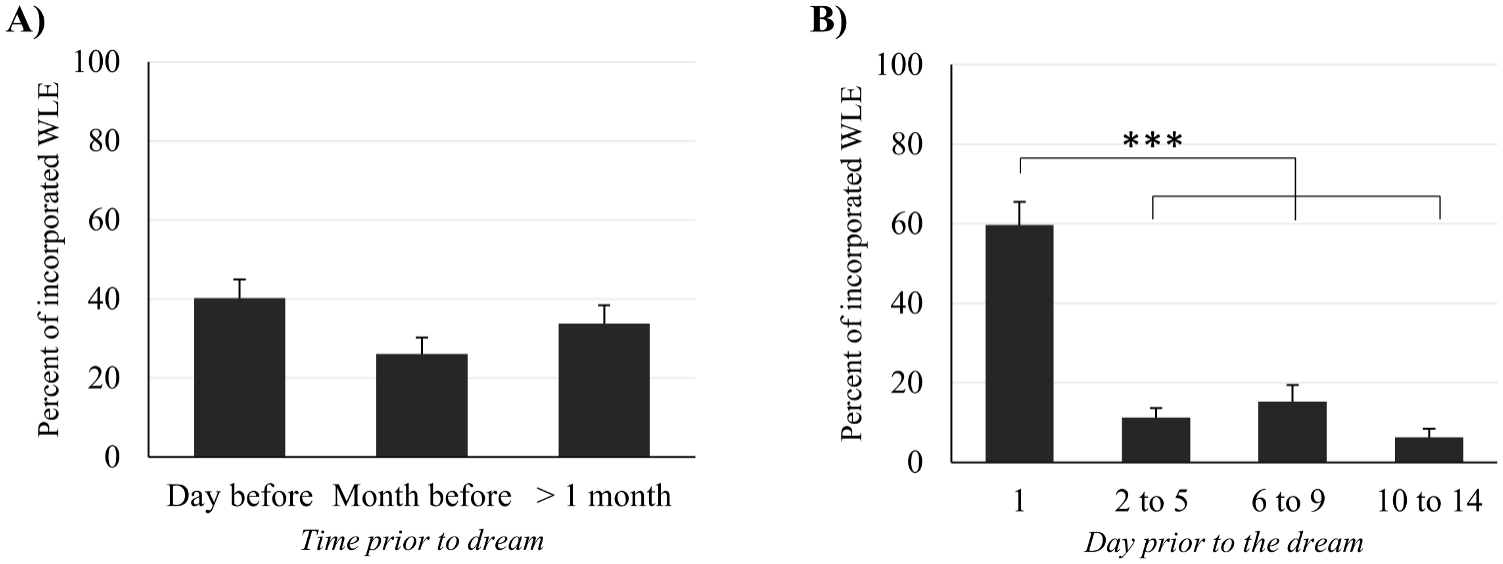
Distribution of the dated WLEs incorporated into dreams as a function of their remoteness. (A) Distribution of all the WLEs incorporated into dreams when 3 categories of remoteness are considered. (B) Distribution of the WLEs incorporated into dreams which happened between 1 day to 14 days when 4 categories of remoteness are considered. Error bars represent standard error of the mean. ***p <.*001*.

In average 79.3 ± 19 percent of the WLEs incorporated into dreams were dated by the participants. Table 3 and Fig 4 show the distribution of dated waking life experiences incorporated into dreams as a function of their date of occurrence in the waking life of the dreamer. Using a one-way repeated measures ANOVA, we observed no significant differences between the proportions of WLEs in the 3 following categories: day before (day-residues), month before (day before the dream excluded) and older than one month. For the analysis focusing on the 15 days before the dreams, a one-way repeated measures ANOVA yielded a significant main effect of time (F(3,39) = 32.3, p <.001). Post-hoc comparisons using the Tukey HSD test indicated that the proportion of WLEs which occurred the day before the dream (59.7% ± 36.9) was significantly larger than the proportions of WLEs that occurred 2 to 5 days (11.2% ± 15.3), 6 to 9 days (15.3% ± 25.8) and 10 to 14 days before the dream (6.3 ± 13.2; p <.001 for all comparisons). No significant differences was found between the other categories. The characteristics (scores averages and distributions) of the WLEs incorporated into dreams that occurred between 6 and 9 days before the dream are presented in S3 Table.

**Table 3:** Percentage of the dated WLEs incorporated into dreams as a function of their temporal remoteness.

	1 day	2 to 5 days	6 to 9 days	10 to 14 days	15 to 31 days	2 to 12 months	More than 1 year
Mean	40.2	7.3	8.1	4.3	6.4	15.8	17.9
SD	30	9	12	9	13	21	24

The time elapsed between the occurrence of the dream and the occurrence of the WLE is used to date WLEs incorporated into dreams. The WLEs from the “1 day” category occurred the day before the dream.

#### Interactions between remoteness and emotion/importance

**Fig 5:**
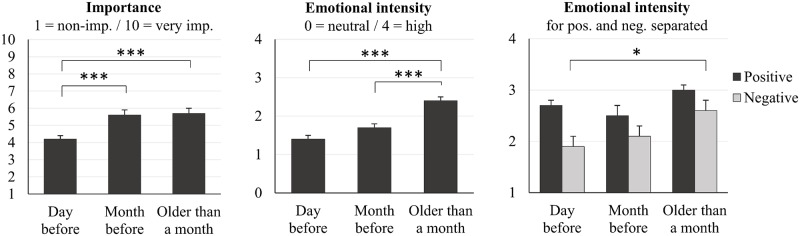
Emotionality of the dated WLEs incorporated into dreams as a function of their remoteness. (A) Importance scores of the WLEs incorporated into dreams as function of their remoteness. (B) Emotional intensity as a function of remoteness. (C) Emotional tone as a function of remoteness. Error bars represent standard error of the mean. *p<.*05*—**p<.*01*—***p<.*001*.

We tested for a possible interaction between remoteness and either emotional tone or emotional intensity or importance. To that purpose, the 492 incorporated WLEs were divided in 3 temporal categories: from the day before, from the month before (day before excluded), and from more than 1 month. For each of the three temporal categories, the means across all incorporated WLEs were computed for importance, emotional intensity and emotional tone (see Table 4 and Fig 5).

**Table 4:** Rating (Mean+SD) of the dated WLEs incorporated into dreams as a function of their remoteness.

	Day before	Month before	Older than a month	*F*	*p*
Importance *1-to-10 scale*	4.2 ± 3.1	5.6 ± 3	5.9 ± 3.1	10.4	.*00*
Emotional intensity *0-to-4 scale*	1.4 ± 1.5	1.7 ± 1.4	2.4 ± 1.4	15.4	.*00*
Positive *1-to-4 scale*	2.7 ± 1.1	2.5 ± 1.1	3 ± 1	1.23	.*29*
Negative *1-to-4 scale*	1.9 ± 1.1	2.1 ± 1.2	2.6 ± 1.1	4.29	.*02*

First, a one-way ANOVA yielded a significant effect of remoteness on the importance ratings of incorporated WLEs (F(2,352) = 10.4, p <.001). Tukey post hoc revealed that incorporated WLEs that happened a month or more before the dream were scored as more important than the ones that happened the day before the dream (respectively 5.6 ± 0.2 vs 4.2 ± 0.2; p = .001 and 5.9 ± 0.2 vs 4.2 ± 0.2; p = .000). Incorporated WLEs that happened during the month preceding the dream (day before the dream excluded) were not rated as more or less important as compared to incorporated WLEs that happened more than one month before the dream (p = .86).

Secondly, there was also a significant effect of remoteness on the emotional intensity rating of the WLEs incorporated into dreams (F(2,389) = 15.4, p <.001). According to Tukey post hoc, incorporated WLEs that happened more than one month before the dream were rated as significantly more intense emotionally (2.4 ± 0.1) than the ones that happened the month before (day before the dream excluded, 1.7 ± 0.1; p <.001) or the day before the dream (1.4 ± 0.1; p <.001)p <.001.

Third, a one-way ANOVA showed a significant effect of remoteness on the rating of the emotional tone of the WLEs incorporated into dreams for the negative subscale (F(2,96) = 3.4, p = .04). Indeed, incorporated WLEs that happened more than one month before the dream were rated as more negative (2.6 ± 0.1) than the ones experienced the day before the dream (1.9 ± 0.1; p = .03). There was no significant effect of remoteness on the rating of emotional tone for the positive subscale.

Finally, to ensure that mundane WLEs did not all date form the day before the dream, the distribution of mundane WLEs incorporated into dreams according to their temporal remoteness was calculated and is presented in S4 Table.

## Discussion

The present study aimed to investigate the characteristics of the waking life experiences that are incorporated into remembered dreams upon morning awakenings at home. Subjects were asked to report and characterize WLEs incorporated into their dreams immediately upon awakening during 7 consecutive days. By contrast with most previous studies, such an approach enabled us to consider WLEs from the whole lifespan of the participants and also mundane ones. Only the main results are discussed below. Discussion of the remaining results is available in S2 File (incorporation of familiar characters and places, and current concerns in dreams).

### The percentage of dreams with incorporation of WLEs

In 84% of all the dreams (N = 247) and in 83% ± 18 of the dreams of each subjects, the dreamer identified at least one obvious waking life source in his or her dream. Asking participants to draw connections between 14-days day and dream diaries, Schredl [10] reported that 41% of 254 dreams had been related to at least one WLE, while in Fosse [1] it was the case for 65% of 299 dreams. Thanks to a survey Botman & Crovitz [7] observed that 83% of 124 dreams incorporated somebody or something from the past while Malinowski and Horton found that 95% of the 186 dream reports contained an element either weakly or strongly related to an autobiographical memory [36]. According to our study, and previous ones which most probably underestimated the percentage of dreams related to waking life features (i.e. either by considering only a small part of the subject’s waking life by using a day diary or asking the subjects to report only the WLEs obviously related to dream content), the incorporation of WLEs into dreams appears to be extremely frequent if not to suppose systematic. Moreover, an average of 1.9 WLE were incorporated per dream in our study which is similar to the results of Marquardt [25] who reported an average of 1.3 WLE per dream using a comparable approach. These results highlight WLEs as a core feature of dream content.

### The emotionality of the WLEs incorporated into dreams

Using a paradigm that takes trivial experiences into account we observed that 46% of all WLEs incorporated into dreams were scored as non-important or neutral by the dreamers and 42.5% as feebly emotionally intense. This result supports our claim that mundane experiences are a significant part of the WLEs incorporated into dreams and that the proportion of emotionally intense WLEs was probably overestimated in previous studies [9,10]. According to our results, trivial WLEs (be it recent or remote, see S4 Table) are largely represented in dreams (S2 Fig).

Coherently with this idea, we found that on average the dreamers scored the WLEs incorporated into their dreams as moderately important and emotionally intense (Table 2). The distribution of the scores show that a slight majority of the WLEs incorporated into dreams were scored as important (i.e. with a score above 5) and as moderately or highly emotionally intense (S2 Table, S2 Fig), as suggested by previous studies [9,10]. However, as we hypothesized, we also observed that the majority of day-residues were scored as feebly emotionally intense and as feebly or neutrally important. This result does justice to Freud who wrote at the end of the 19th century in his book the Interpretation of Dreams [24] (http://psychclassics.yorku.ca/Freud/Dreams/dreams.pdf, p.176, emphasis added) “*Although the foregoing remarks have restricted the significance of the* *day-residues* *for the dream*, *they are none the less deserving of some further attention*. *For* *they must be a necessary ingredient in dream-formation*, *inasmuch as experience reveals the surprising fact that* *every dream shows in its content a connection with a recent waking impression*, *often of the most indifferent kind*”.

According to the hypothesis of a preferred incorporation of emotionally intense experiences into dreams [9,10], one would expect to observe more important and emotionally intense WLEs than trivial ones among day-residues. As a consequence, our results question the hypothesis that important and emotionally intense waking life experiences are generally more represented in dreams than are trivial ones.

Regarding the valence of the WLEs incorporated into dreams we observed that a large majority were scored as positive or neutral and that less than 30% were reported as negative. Interestingly, we also found that the valence of the dreamt version of the WLEs was on average experienced as less positive than in waking life. Separate analyses for positive, negative and neutral WLEs further revealed that the valence of the dreamt version of the WLEs was experienced as less positive than in waking life for positive WLEs, as less negative than in waking life for negative WLEs and as as neutral as in waking life for neutral WLEs. The possible implications for dream functions are further discussed below.

### The temporal remoteness of the WLEs incorporated into dreams

Among all the dated WLEs incorporated into dreams we found that 40% were day residues i.e. from the day before the dream (Fig 4A). This is very similar to the 44% reported by Marquardt [25] using a similar procedure as ours but it is widely lower than the 94% reported by Hartmann [18] after he analyzed 800 of his own dreams. Using a different approach (a survey comprising 340 students), Botman & Crovitz [7] reported that 63% of the 103 dreams which had incorporated WLEs, presented a day-residue. All together these results highlight a very important influence of the day before the dream on dream content, as observed by Freud a century ago [24] (see quotation above). However, this study has also highlighted, as hypothesized, the large participation of remote WLEs in dream content. WLEs which happened more than one month before the dream represent approximately one third of all the WLEs incorporated into dreams, and those that happened more than one year before the dream represent nearly 20% (Fig 4A and Table 3).

Regarding the dream lag effect (S3 Table) we found that the percentage of WLEs that happened 6 to 9 days prior to the dreams was not significantly different from the percentages of WLEs that happened 2 to 5 days or 10 to 14 days prior to the dreams. These results question the existence of a dream lag effect in our data.

A remarkable finding of this study is the significant interactions that we have found between temporal remoteness and each of importance, emotional intensity and valence (Fig 5). One can see on Fig 5A and 5B that, as we hypothesized, day residues were scored significantly lower than older WLEs incorporated into dreams on the importance and the emotional intensity scales. In addition the day residues were scored as less negative than the oldest incorporated experiences (Fig 5C). In other words, the oldest incorporated memories were found to be more emotionally intense, more important and more negative than day residues. Interestingly, this effect was also partially reported by Hartmann [18] who found that *“the relatively rare earlier waking experiences from more than 1 day before the dream were more likely to be important*.*"* These results raise several questions with regards to the current hypotheses about dream function.

### Implication for the hypothesis of a role of dreaming in emotional regulation

A current mainstream hypothesis proposes that dreams participate in emotional regulation, through an active moderation of waking life affects [27,28,37–39]. If so, one may expect 1) that dreams incorporate more emotional experiences than non-emotional experiences, 2) that the majority of incorporated WLEs have a negative valence, 3) that the most negative WLEs incorporated into dream date from recently before the dream. Our data do not verify the first prediction since 73% of the WLEs incorporated into dreams were rated as feebly or moderately emotionally intense. The second prediction is not verified either since 72% of the WLEs incorporated into dreams were neutral or positive. This result is coherent with previous ones showing that the average valence of WLEs incorporated into dreams is positive and not significantly different from that of WLEs not incorporated into dreams [10]. Finally, the third prediction is also overruled by our results since we found that the most negative WLEs incorporated into dreams were also the oldest ones. A precaution needs however to be taken regarding this result since it may be at least partly explained by the relatively rare frequency of very negative events in the lives of our participants (young healthy French students) and by the short duration of the study (7 days). Given that our results did not support the 3 above predictions, they may, as a consequence, be interpreted against a role of dreaming in emotional regulation. However an alternative interpretation could be that our predictions were inadequate and that dreaming may participate in emotional regulation through another mechanism.

Interestingly, although 45% of the WLEs incorporated into dreams had a positive valence, 40% of dreams were found to have a negative emotional tone. Even more interesting, we found that in average, for positive and negative WLEs, the emotional tone of the dreamt version of the WLE was rated as less intense than its original version (Fig 1A). Positive WLEs were less positive in the dream, negative WLEs were less negative in the dream and neutral WLEs remained neutral in the dream. Dreams seem thus to attenuate the emotional intensity of emotional memories and somehow modulate their emotional tone towards a more neutral one. This effect cannot be explained by a general effect of moderation of emotional intensity in dreams since the range of the emotional gradation of the dream content was maximum (range: 1–10). It is also important to note that participants rated the emotional valence of the original WLE and its dreamt version at the same time, the intensity difference cannot thus be explained by a delay between the two notations. The very significant correlation between the emotional grades of the original WLEs and the emotional grades of the dreamt WLEs further shows that the dreams linearly decrease the emotional intensity of WLEs towards neutrality (Fig 1B). The dreams even seem to normalize the distribution of emotional grades, since the distribution of the grades of the original WLEs is dissymmetric (higher frequency of very positive than very negative WLEs, Fig 1B) whereas the distribution of the grades of the dreamt version is not. Our results suggest that dreams participate to emotional regulation through a relative neutralization of the emotional intensity of both negative and positive WLEs incorporated into dreams. The gradation of similarity between the original version and the dreamt version of the WLEs shows that the original WLEs are recognizable but transformed (average gradation ~ 6/10, Fig 3). The dream seems thus to keep the WLE recognizable and to also modify it (e.g. changing the context, the plot) just enough to diminish its emotional intensity. This mechanism may have a significant impact since the majority of the WLEs incorporated into dreams are important for the dreamer, a lot are of high emotional intensity (mean percentage of WLEs with an emotional rating > 8 or < 3 = 31.5 ± 23.7%) and a significant proportion are concerns (S2 Fig) of the daily life (e.g. work-, relatives-, love- related, S1 Fig). These findings are the first strong experimental arguments in favor of the emotional regulation hypothesis of dreaming, and are consistent with the numerous experimental results showing a modulation of affective neural systems and the (re)processing of recent emotional experiences during sleep [40,41]. Interestingly, our results suggest that the mechanism by which the emotional regulation is made during dreaming does not require that the dream is recalled to be efficient i.e. they suggest that whether or not the dream is recalled, the dreamt WLE is experienced with diminished emotional intensity.

### Implication for the hypothesis of a role of dreaming in memory consolidation

Some researchers have suggested that dreaming reflects the physiological process of consolidating novel memories and assimilating them into a large memory network [29–31,42]. One proposed mechanism is that emotional intensity is the parameter triggering the selection of memories for consolidation into long term memory [9,43]. In our data, the most emotionally intense experiences incorporated into dreams were the oldest ones and day residues were rather trivial (with low scores of importance and emotional intensity). This result does not really fit with the idea of emotional intensity tagging the recent memory traces to be consolidated during sleep. In addition the WLEs incorporated into dreams were scored as rather familiar, and they were rarely repeatedly incorporated into dreams across the week. This result comes against the hypothesis that dreaming consolidates novel memory traces. An alternative hypothesis might be that dream content reflects the physiological down-regulation of recent useless memories [44,45]. This would explain the great proportion of day residues of low importance and low emotional intensity in dream reports. In the light of the above discussion, the link between dreams and memory consolidation is still unclear and would need further testing. Notably, the role of mundane day residues would need to be better understood and taken into account in a memory consolidation model of dreaming.

## Conclusion

Important contributions of this study are to provide significant arguments in favor of the emotional regulation hypothesis of dreaming and to show the importance of day residues in dream content and their tendency to be mundane. In addition, our results clearly show that dreams mix various and opposite elements of waking life which are all incorporated in significant proportions, i.e. recent and old, emotionally loaded and emotionless, positive and negative, rare and occurring daily, familiar and new, important and insignificant, concerns and non-concern issues.

A possible explanation of the heterogeneity and diversity of WLEs incorporated into dream content could be that dreams are an open window on the cognitive processes taking place during sleep. According to our current understanding, sleep is indeed involved in various cognitive processes such as memory consolidation, forgetting, emotional regulation, creativity and stimuli processing [40,41,44,46–50].

One may speculate according to our results and previous ones that sleep classifies and reprocesses mainly the information processed during the day before, while also processing the incoming information. Indeed a great part of the WLEs incorporated into dreams are from the day before the dream. Some of the remaining ones may have been considered as more remote because participants forgot that they thought about these WLEs the day before the dream (thoughts may be more difficult to recall than actions and perceptions) and the other remaining ones may be related to the day-residues, as already proposed by Marquardt [25]. Dreams would thus be the witnesses of sleep working at reprocessing what has been processed during the previous day, consolidating some information, incorporating it in a larger network of memories, forgetting other information, regulating emotions, promoting creativity and processing stimuli all together. If so, it would explain the great heterogeneity and lack of coherence of dream content mixing all together information to be erased or consolidated, information to be down-regulated at the emotional level and even old memories to be related to newly consolidated information. Emotion could in this case be the tag which decides how each items of information is processed during sleep. The strength of this proposition is to encompass and explain most of the characteristics of dream content and not only some of them, as it has been the case for previous hypotheses until now (see [5] for a review).

## Limitations

This study aimed at investigating dream content obtained in naturalistic conditions which implied that participants slept at home, without electroencephalographic recordings, and that they had to report one dream per night in the morning. As a consequence we have investigated only dreams from the end of the sleep period, and our results apply only to this type of dream. Results may have been different if we had collected dreams from the beginning of the night. Indeed, some studies have shown that the remoteness of memory sources of dreams may increase from the beginning to the end of the night [13,14]. However other studies suggest a continuity in the thematic content of dreams across the night [51,52].

Given our protocol we do not know which sleep stage dreams occurred in. However, there is an approximately equal likelihood that morning awakenings occur in REM or N2 sleep [53], and the differences between the contents of N2 and REM sleep dreams become less marked in the second half of the night [54]. As a consequence, it seems likely that our results are valid for both N2 and REM sleep dreams of the end of the night.

However, as previous studies have shown that the mean percentage of episodic memory sources is significantly greater for NREM than for REM dreams whatever the time of the night [55], it would be worth testing in future studies whether the results presented here can be reproduced for NREM and REM dreams separately.

Note that the protocol was not designed to dissociate episodic, autobiographic and semantic memories among the memory sources of the dream [36,55,56]. This point would be worth investigating in future studies, carried out with explicit rules to be applied by external judges to classify type, emotionality and remoteness of dream memory sources.

## Supporting information

S1 Table Examples of waking life elements incorporated into dreams. (DOCX)

S2 Table Distribution of the score given to WLEs incorporated into dreams, for all WLEs (bold) and day-residues only. (DOCX)

S3 Table Characteristics of the WLEs incorporated into dreams that happened 6 to 9 days before the dreams (n = 36). (DOCX)

S4 Table Distribution (%) of mundane WLEs incorporated into dreams according to their temporal remoteness. (DOCX)

S1 Fig Concerns reported in the initial questionnaire. Concerns were distributed in 7 thematic categories. The number of concerns per categories are represented in pink. Red bars illustrate the percentage of concerns from one category that were incorporated into dreams during the 7-days experiment.
(TIF)

S2 Fig Distribution of the scores for all the WLEs incorporated into dreams and day-residues only (see S2 Table for the figures). (TIF)

S1 File Supp_discussion. (DOCX)

S2 File Supp_results. (DOCX)
