# Correction: Characteristics of the memory sources of dreams: A new version of the content-matching paradigm to take mundane and remote memories into account

**DOI:** 10.1371/journal.pone.0193440

**Published:** 2018-02-21

**Authors:** Raphael Vallat, Benoit Chatard, Mark Blagrove, Perrine Ruby

## Notice of republication

This article was republished on October 27, 2017 to replace incorrect versions of S1 Table and S3 Table. Please download this article again to view the corrected tables.
