# Supplementary material for: Characteristics of the memory sources of dreams: A new version of the content-matching paradigm to take mundane and remote memories into account
Source: PLoS One. 2017 Oct 11;12(10):e0185262. doi: 10.1371/journal.pone.0185262 (PMC5636081; doi:10.1371/journal.pone.0185262)
Supplement: S1 Table — (DOCX) [file pone.0185262.s001.docx]

**Old, important and emotionally negative WLE**

S1 | Valence = 2 ; Importance = 7 ; Dream valence = 5

Dream report: *"I am with an ex-girlfriend in my dream, we get along together very well"*

WLE description: *"We broke up several years ago in a difficult situation and none of us had given sign of life since."*

S29 | Valence = 2 ; Importance = 10 ; Dream valence = 1

Dream report: *“I descend into another world to pick up my aunt and realize that it is not possible - I feel a deep anguish and I am cold”*

WLE description: “*My aunt passed away 3 months ago and I miss her terribly. I know she will not come back, but some days I still hope it will happen.”*

S35 | Valence = 2 ; Importance = 10 ; Dream valence = 3

Dream report: *“In my dream I saw my ex-girlfriend and her new partner. Suddenly, I felt really angry and started to push them down the stairs. They fell down and I shout at them."*

WLE Description: *"Three years ago I bumped into them in the streets and was particularly unkind to them."*

**Old, important and emotionally positive WLE**

S6 | Valence = 10 ; Importance = 10 ; Dream Valence = 10

Dream report: *"I was comforting two twins sisters that just got fired from the religious association I was working in several years ago."*

WLE description: *"It reminded me of the responsibility I used to have there."*

S19 | Valence = 10 ; Importance = 10 ; Dream Valence = 10

Dream report: *"I am working at my shop with my employee and my friend F* with whom I get along very well"*

WLE description: *"F* is an old school friend. At the time we were always together and for me he was almost part of the family"*

**Mundane and feebly emotional day-residues**

S3 | Valence = 5 ; Importance = 1 ; Dream valence = 6

Dream report: *"I was in an unknown house with two friends and a talking Koala.”*

WLE description: “*It reminded me of the talking raccoon in the movie Guardian of the Galaxy that I watched the day before. "*

S15 | Valence = 5 ; Importance = 1 ; Dream valence = 5

Dream report: *"I am in the supermarket looking for a dishwasher."*

WLE description: *"Yesterday I saw on the internet a picture of the proper way to load dishes in a dishwasher."*

S25 | Valence = 5 ; Importance = 1 ; Dream valence = 5

Dream report: *"In my dream I was creating magical creatures in order to destroy something”.* WLE description: “*The creatures reminded me of the ones in the video game I played for several hours the day before."*

S38 | Valence = 5 ; Importance = 1 ; Dream valence = 5

Dream report: *"I was in a house with a small swimming-pool.”*

WLE description: “*The swimming-pool was very similar to the one I saw yesterday in a park. The shape, depth and color were the same.*

**Important and emotionally intense day-residues**

S40 | Valence = 1 ; Importance = 10 ; Dream Valence = 1

Dream report: *"I was at work, struggling to fix several mistakes made by my boss. At the end of the day, I took the blame and got fired."*

WLE description: *"Yesterday there was a problem in my company after a client requested a sudden change."*

S33 | Valence = 2 ; Importance = 8 ; Dream Valence = 4

Dream report: *"My father offers me a sewing machine. I realize that I already have this model."*

WLE Description: *"My father is a recurring concern and we talked yesterday about his health. Also, yesterday I thought that I should sew this week-end."*

S29 | Valence = 10 ; Importance = 10 ; Dream Valence = 10

Dream report: *"I was talking to several persons at the university and each time I tried to say only positive things in order to make them happy."*

WLE description: *"Yesterday I thought that I should really stop being always negative and doubtful. In my dream it felt as if I put into practice this."*

S19 | Valence = 10 ; Importance = 10 ; Dream Valence = 10

Dream report: *"In my dream there was this cartoon character that I really like."*

WLE description: *"Yesterday I watched a really good new cartoon movie."*
