# Supplementary material for: Characteristics of the memory sources of dreams: A new version of the content-matching paradigm to take mundane and remote memories into account
Source: PLoS One. 2017 Oct 11;12(10):e0185262. doi: 10.1371/journal.pone.0185262 (PMC5636081; doi:10.1371/journal.pone.0185262)
Supplement: S2 Table — (DOCX) [file pone.0185262.s002.docx]

| Characteristics | Low *(%)* | Neutral (%) | High *(%)* |
| --- | --- | --- | --- |
| Frequency *(Rare – Daily)* | **53.5 ± 22.3** | **12.4 ± 11.4** | **34.2 ± 24.2** |
|  | 52.1 ± 33 | 16.2 ± 25 | 31.7 ± 35 |
| Familiarity *(New – Familiar)* | **33.6 ± 21.7** | **9.9 ± 14.5** | **56.5 ± 23** |
|  | 43 ± 37 | 14.2 ± 25 | 42.9 ± 37 |
| Emotional valence *(Neg. – Pos.) ^1^* | **28.1 ± 20.9** | **26.6 ± 25.3** | **45.3 ± 26.2** |
|  | 23.9 ± 28 | 35.9 ± 34 | 40.2 ± 37 |
| Importance | **34.4 ± 23.6** | **12 ± 12.7** | **53.5 ± 22.9** |
|  | 48.2 ± 37 | 12.2 ± 25 | 39.6 ± 38 |
| Current concern | **56.6 ± 25.1** | **9 ± 13.8** | **34.4 ± 21.6** |
|  | 57.8 ± 37 | 9.1 ± 21 | 33.1 ± 33 |
| Emotional intensity ^2^ | **42.5 ± 25.4** | **32.9 ± 24.4** | **24.6 ± 23.4** |
|  | 52.6 ± 38 | 24.9 ± 32 | 22.5 ± 33 |

S2 Table. Distribution of the score given to WLEs incorporated into dreams, for all WLEs (bold) and day-residues only.

^1^ For emotional valence, Low = negative and High = positive.

^2^ Emotional intensity is rated on a 1-to-4 scale (see Methods). Neutral = medium emotional intensity.
