# Supplementary material for: Characteristics of the memory sources of dreams: A new version of the content-matching paradigm to take mundane and remote memories into account
Source: PLoS One. 2017 Oct 11;12(10):e0185262. doi: 10.1371/journal.pone.0185262 (PMC5636081; doi:10.1371/journal.pone.0185262)
Supplement: S3 Table — (DOCX) [file pone.0185262.s003.docx]

S3 Table. Characteristics of the WLEs incorporated into dreams that happened 6 to 9 days before the dreams (n=36).

| Characteristics | Mean Score ± SD | Low (%) | Neutral (%) | High (%) |
| --- | --- | --- | --- | --- |
| Frequency *(Rare – Daily)* | 4.1 ± 2.9 | 49.3 ± 48 | 18.6 ± 37 | 32.1 ± 42 |
| Familiarity *(New – Familiar)* | 5.2 ± 3.0 | 38.1 ± 43 | 25.2 ± 42 | 36.7 ± 42 |
| Emotional valence *(Neg. – Pos.) ^1^* | 5.4 ± 2.1 | 24.6 ± 36 | 22.5 ± 39 | 52.9 ± 46 |
| Importance | 5.7 ± 3.0 | 21.9 ± 34 | 19.5 ± 31 | 58.6 ± 39 |
| Current concern | 5.1 ± 3.4 | 39.3 ± 40 | 8.6 ± 27 | 52.1 ± 40 |
| Emotional intensity *^2^* | 1.4 ± 1.3 | 56.1 ± 46 | 31.8 ± 41 | 12.1 ± 29 |

Except for emotional intensity, Neutral (%) refers to the percentage of WLEs with a score of 5.

^1^ For emotional valence, Low = negative and High = positive.

^2^ Emotional intensity is rated on a 1-to-4 scale (see Methods). Neutral = medium emotional intensity.
