# Supplementary material for: Characteristics of the memory sources of dreams: A new version of the content-matching paradigm to take mundane and remote memories into account
Source: PLoS One. 2017 Oct 11;12(10):e0185262. doi: 10.1371/journal.pone.0185262 (PMC5636081; doi:10.1371/journal.pone.0185262)
Supplement: S4 Table — (DOCX) [file pone.0185262.s004.docx]

S4 Table. Distribution (%) of mundane WLEs incorporated into dreams according to their temporal remoteness.

|  | Day before | Month before | More than a month | Not dated |
| --- | --- | --- | --- | --- |
| Importance < 5, *n=196* | 43.4 | 19.9 | 20.4 | 16.3 |
| Importance = 1, *n=96* | 52.1 | 17.7 | 17.7 | 12.5 |
| Importance = 1 and Emotional intensity = Low, *n=70* | 60 | 20 | 8 | 12 |

Each time category mutually excludes the previous ones (e.g. month before excludes day before the dream).
