# Supplementary figures and images for: Characteristics of the memory sources of dreams: A new version of the content-matching paradigm to take mundane and remote memories into account
Source: PLoS One. 2017 Oct 11;12(10):e0185262. doi: 10.1371/journal.pone.0185262 (PMC5636081; doi:10.1371/journal.pone.0185262)

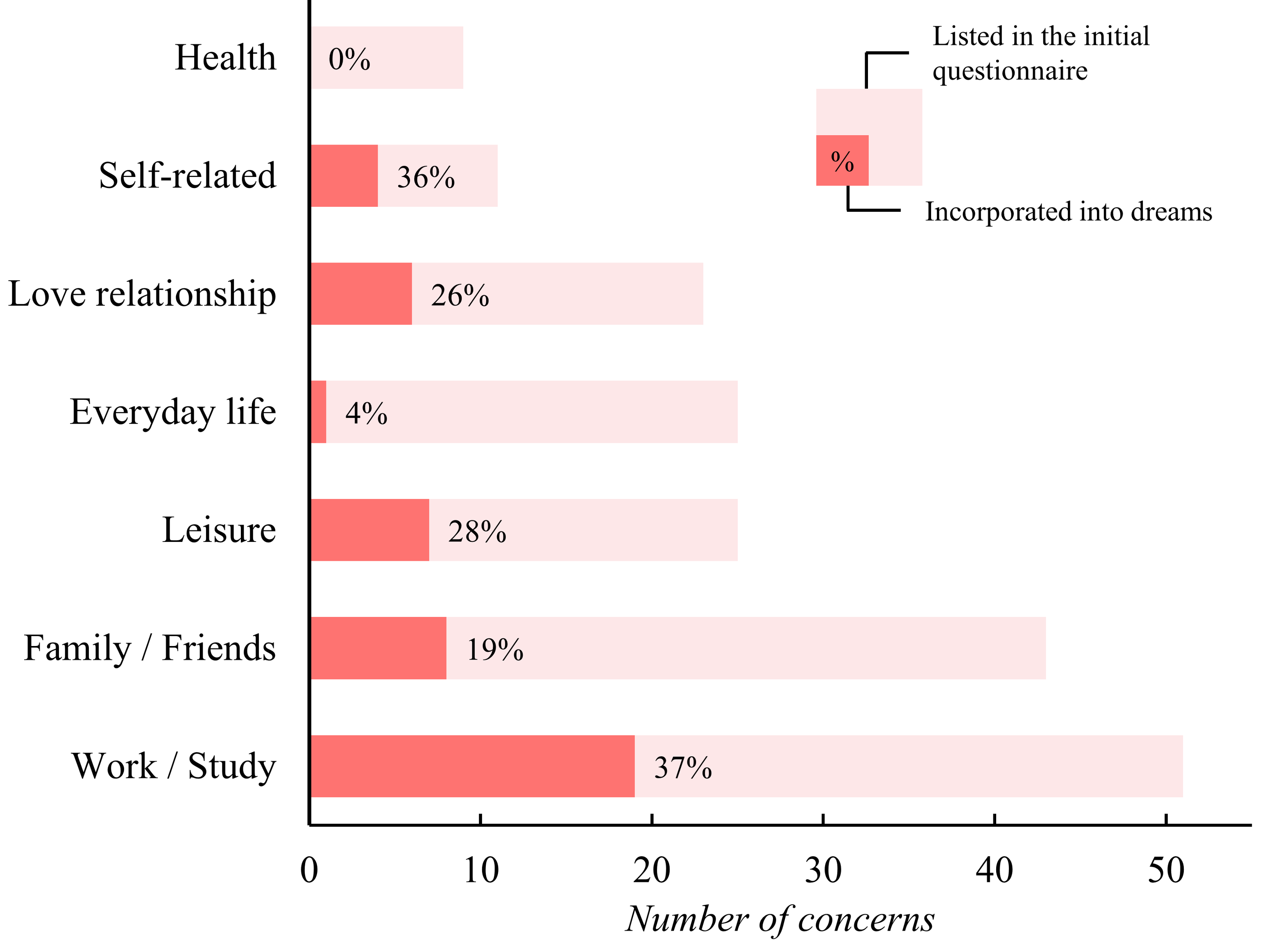

Supplement: S1 Fig — Concerns were distributed in 7 thematic categories. The number of concerns per categories are represented in pink. Red bars illustrate the percentage of concerns from one category that were incorporated into dreams during the 7-days experiment. (TIF) [file pone.0185262.s005.tif]

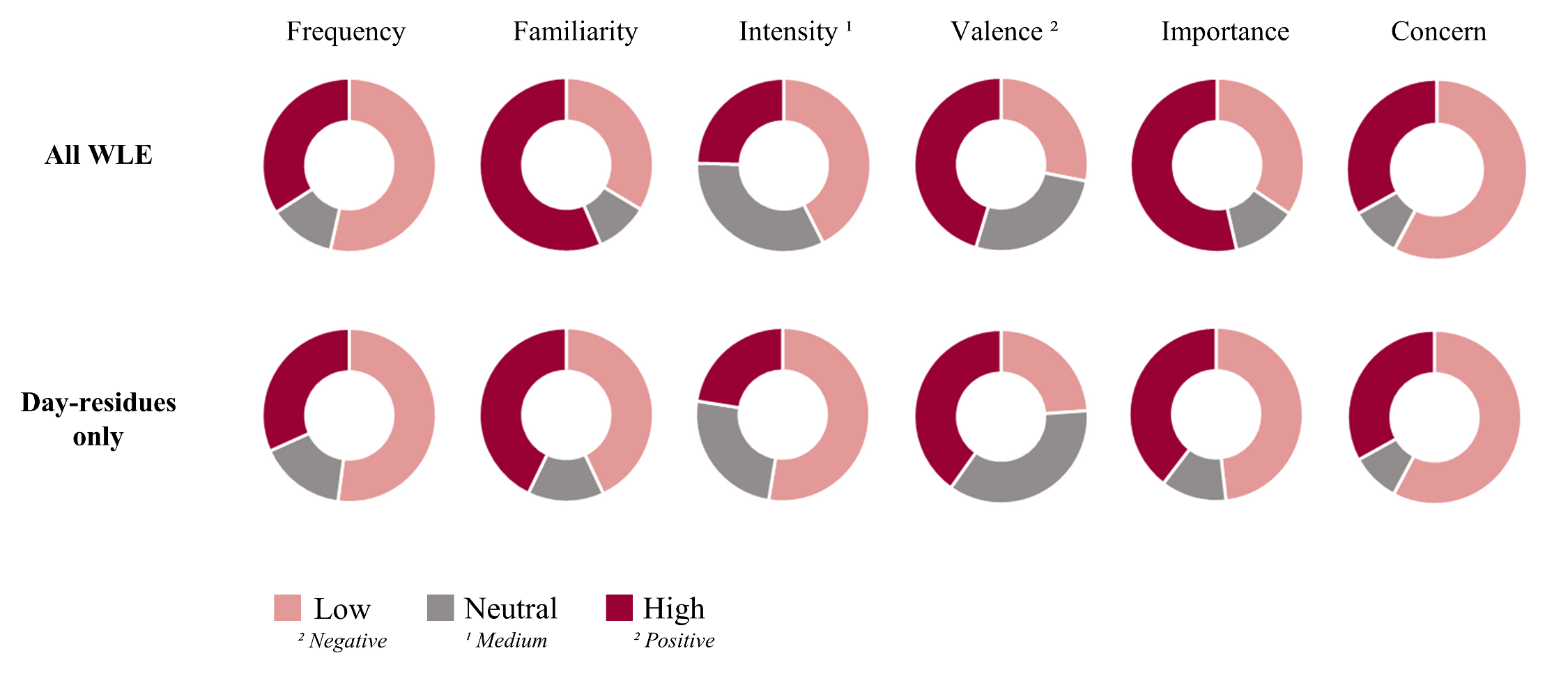

Supplement: S2 Fig — (TIF) [file pone.0185262.s006.tif]
