# Supplementary material for: Characteristics of the memory sources of dreams: A new version of the content-matching paradigm to take mundane and remote memories into account
Source: PLoS One. 2017 Oct 11;12(10):e0185262. doi: 10.1371/journal.pone.0185262 (PMC5636081; doi:10.1371/journal.pone.0185262)
Supplement: S1 File — (DOCX) [file pone.0185262.s007.docx]

## The concern-related dimension of WLEs incorporated into the dreams

According to the dreamers’ scoring, the WLEs incorporated into dreams are not predominantly concern-related (Table 2). This result is coherent with the experimenters’ assessment of the incorporation of current concerns into dreams taking the initial questionnaire into account. We found that in average 23% of the dream reports of each subjects incorporated a current concern. Reciprocally, on average for each subject only 25% of the concerns listed in the initial questionnaire were incorporated into a dream report during the 7 days of the experiment. Previous studies reported a larger percentage of dreams in relation with the dreamers’ current concerns. For example, Schwartz & Maquet [1], who used an automatic analysis of words on 1770 dreams of the first author found that 35% of the dreams were considered related to current concerns. The great heterogeneity between studies may come from different definitions of the term “concerns” and from different methods (if close friends and family were considered as concerns, the % of dreams related to concerns would be much higher in our study). Our results show that when listed a priori (i.e. before dreams content analysis), current concerns are not as represented in dreams as would be expected from the dominant hypothesis saying that “much of it tends to revolve around a relative handful of personal concerns” [2].

## The characters and places incorporated into the dreams

At least one external character was reported for nearly all remembered dreams. The mean number of characters per dream was slightly above with the norm (2.6 in Hall & Van de Castle [3]). This results is most likely due to several methodology differences: in Hall & Van de Castle system, rating was done *a posteriori* by an external judge, who would consider groups of characters not individually named (e.g. a couple, a group of 3 children) as one character. In our study, the number of characters was assessed by the dreamer and considering each characters of the dreams as one individual. Regarding familiar existing persons (Fig 1) our results even if a little higher, are also coherent with the norm (familiar characters, 45% in males & 58% in females in Hall & Van de Castle [3]). However, in our data close family and friends appeared to be more represented than in the normative study (family, 9% in males, 14% in females; relatives, 2% in males, 4% in females in Hall & Van de Castle [3])

Regarding places, in our study participants reported nearly twice as much places (2.3 ± 1.5) as in the norm (average n° of settings per dream, 1.3 in Hall & Van de Castle [3]) and nearly half of these places were unknown (Fig 1) which is far more than the norm (unfamiliar, 14% [3]). Finally, we observed a little less familiar places (familiar, 33% [3]) and a little less mixed places (questionable, 40% [3]). It is important to keep in mind that in our study the dreamers rated their own dreams while in Hall & Van de Castle [3] an external rating was used. The two methods may yield divergent results [4].

## References

1. Schwartz S, Maquet P. Sleep imaging and the neuro-psychological assessment of dreams. Trends Cogn Sci. 2002;6: 23–30.

2. Domhoff GW, Schneider A. Studying dream content using the archive and search engine on DreamBank.net. Conscious Cogn. 2008;17: 1238–1247. doi:10.1016/j.concog.2008.06.010

3. Hall CS, Van de Castle RL. The content analysis of dreams. 1966;

4. Sikka P, Valli K, Virta T, Revonsuo A. I know how you felt last night, or do I? Self- and external ratings of emotions in REM sleep dreams. Conscious Cogn. 2014;25: 51–66. doi:10.1016/j.concog.2014.01.011
