# Supplementary material for: Characteristics of the memory sources of dreams: A new version of the content-matching paradigm to take mundane and remote memories into account
Source: PLoS One. 2017 Oct 11;12(10):e0185262. doi: 10.1371/journal.pone.0185262 (PMC5636081; doi:10.1371/journal.pone.0185262)
Supplement: S2 File — (DOCX) [file pone.0185262.s008.docx]

**Distribution of the scores**

For each characteristic, the percentage of WLEs with a rating inferior, equal and superior to 5 was computed. A visual inspection of Table S2 shows that if we compare the distribution of the scores for all the WLEs incorporated into dreams and day-residues only, the distributions differ for familiarity, importance, and emotional valence and intensity. As compared to all WLEs incorporated into dreams, for day-residues only we observed a greater percentage of WLEs scored as feebly familiar, emotionally neutral, feebly important and feebly emotionally intense (S2 Fig).
